# An efficient three-tier defense mechanism for mitigation of DDoS attack with port connection analysis in SDN

**DOI:** 10.1038/s41598-025-33463-z

**Published:** 2026-01-12

**Authors:** Anam Rajper, Norlina Binti Paraman, Muhammad Nadzir Marsono, Noor Jahan Rajper, Hira Hameed, Muhammad Usman

**Affiliations:** 1 Faculty of Electrical Engineering, Universiti Teknologi Malaysia, Johor Bahru, 81310 Skudai Malaysia; 2 Institute of Mathematics and Computer Science, University of Sindh, Jamshoro, 76080 Sindh Pakistan; 3 Department of Computing, Imperial College London, SW7 2AZ London, UK; 4 School of Science and Engineering, Glasgow Caledonian University, G4 0BA Glasgow, UK

**Keywords:** Engineering, Mathematics and computing

## Abstract

This study presents a novel three-tier defense mechanism at the software-defined networking (SDN) control plane to improve DDoS attack detection and mitigation using real-time flow table data from OpenFlow switches. The proposed method combines adaptive statistical detection, event-based activation of the ML classifier, and targeted port-level mitigation to increase accuracy and lower controller load, in contrast to current solutions that rely on static thresholds, full attack-path tracing, or continuously running machine learning models. The proposed model employs an improvised cumulative sum algorithm at the first tier with adaptive threshold and an event-based activation of decision tree classifier at second tier to swiftly and accurately detect DDoS traffic with sub-second latency without adding extra load on SDN controller. The third tier uses port connection analysis by utilizing link-layer discovery protocol (LLDP) that distinguishes direct and indirect port sources involved during attack without tracing the complete attack path. Results demonstrate that this integrated mechanism offers faster, more precise, and more resource-efficient DDoS mitigation compared with existing solutions. It outperforms traditional methods by achieving accuracy of 99.99%, reducing computational load and false positive rate by 87%, and achieve a more targeted response by reducing unnecessary mitigation actions by 94% with 0% packet loss.

## Introduction

Software-defined networking (SDN) is one of the new modern networking technologies that has introduced flexibility, programmability, and scalability in networking by decoupling the control plane from its data plane. This new paradigm has introduce complete centralized control over network management with optimizes resource utilization while dynamically adapting to changing network conditions^1^. Although SDN is a modern networking paradigm, one of its key weaknesses is also its centralized control architecture, which makes it more vulnerable to Distributed Denial of Service (DDoS) attacks than conventional networks. This type of attack floods the SDN controller or communication channel between the controller and switches with fake requests, causing significant disruptions in the networks or complete loss of network services^2^. The growing incidence and complexity of DDoS attacks further complicate the defense of SDN.

Several research studies have proposed different defense mechanisms with different implementation approaches, each of which has its own specific advantages and disadvantages. Many recent works have used machine learning (ML) and deep learning (DL) models for DDoS detection within an SDN framework. However, most of these models require huge training data along with highly expensive computational methods, which not only overload the controller but also make it difficult to detect DDoS attacks in real time. These models have high false positive rates that may lead to unnecessary mitigation actions and consequently downgrade the quality of the network. While alternative statistical approaches (such as entropy and cumulative-sum) are much faster, they are also less adjustable due to their dependence on fixed thresholds, which is not suitable for the dynamic nature of traffic patterns.

Traditional DDoS mitigation techniques (for examples, filtering, rate limiting, access controls, and port scanning) neither offer targeted mitigation nor fully utilize the programmability and flexibility of SDN. This limitation often tends to result in a pervasive block of valid and malicious traffic, deteriorating overall network performance. Existing SDN defense mechanisms generally fall into five categories: traffic-management enforcement, access-control mechanisms, real-time monitoring and policy adaptation, statistical detection, and machine/deep-learning approaches. The following subsections highlight three main limitations across existing traditional solutions: 1) Coarse-level mitigation that disrupts legitimate traffic, 2) issues of latency scalability, and 3) computationally expensive mechanisms leading to delays in mitigation response, as referred to in Tables 1, 2 and 3.

**Table 1:** Startegy used and limitation of existing defense mechanism based on their categories: traffic management and access control.

**Category**	**Approach**	**Description**	**Key limitations**
Traffic Management	Connection Management (Shin et al.^3^)	utilized connection management and trigger activation to prevent unauthorized access ahead of time.	Their method does not differentiate between individual switch levels, which causes legitimate traffic to be unnecessarily blocked.
	FloodGuard (Wang et al.^4^)	presented FloodGuard that includes proactive flow rule installation and packet migration for dynamic traffic control.	Although this technique is effective, it applies rate-limited packet processing indiscriminately and could thus degrade service quality to legitimate users due to huge volumes of traffic.
	FlowFense (Piedrahita et al.^5^)	proposed FlowFense which constrains bandwidth upon detection of congestion.	This method has the potential to penalize any traffic passing through affected pathways, even legitimate traffic.
	SDN-Guard (Dridi et al.^6^)	proposed SDN-Guard, which dynamically reroutes traffic and adjusts flow timeouts to mitigate threats.	However, it lacks detailed flow-specific analysis, causing unnecessary rerouting in unaffected network segments.
Access Control	Flow Tracking (Wang et al.^7^)	focused on stringent access controls and network flow tracking to enhance security.	Though, their approach requires frequent updates and can delay legitimate traffic.
	Peer Support (Yuan et al.^8^)	utilized a peer support strategy to redistribute processing loads and manage flow table resources.	But, this technique overlooks the attack’s origin, leading to potential inefficiencies in resource distribution.
	ArOMA (Sahay et al.^9^)	proposed ArOMA, which facilitates automated mitigation actions between customers and ISP controllers.	However, this centralized policy generation can lead to delays and create a single point of failure.
	C2C Protocols (Hameed et al.^10^)	advocated for Controller-to-Controller communication protocols to expedite attack information distribution for swift, collaborative defense.	However, this method may neglect the detailed data provided by individual switch flow tables, potentially causing inefficiencies in response times and mitigation efforts.
	SGS Framework (Wang et al.^11^)	introduced the SGS framework for workload distribution and targeted traffic management.	But, it could potentially simplify DDoS threat responses by not fully utilizing flow table data.

**Table 2:** Startegy used and limitation of existing defense mechanism based on their category: real-time monitoring.

**Category**	**Approach**	**Description**	**Key limitations**
Real-time Monitoring	Selective Blocking (Conti et al.^12^)	proposed strategies involving selective blocking and periodic monitoring to adapt to real-time attacks.	While effective in reducing the attack impact, these strategies could inadvertently block legitimate traffic, especially when relying on less granular data.
	Policy Storage (Karmakar et al.^13^)	emphasized the importance of policy specification and storage via northbound applications to secure traffic flows.	But, this reliance on rapid policy application may result in delays and scalability issues as networks expand.
	Dynamic Configuration (Mishra et al.^14^)	proposed a dynamic approach to network configurations by taking advantage of centralized control potential of SDN’s.	However, in scenarios that require rapid reconfigurations and constant monitoring, this method faces limitations that may not be feasible in all network conditions.

**Table 3:** Structured comparison of SDN DDoS defense mechanism based on category of statistical methods and ML/DL approaches.

**Category**	**Approach**	**Description**	**Key limitations**
Statistical Methods	DOCUS (Shalini et al.^15^)	introduced DOCUS, a statistical based approach, focuses on isolating the attack vectors by blocking traffic at their respective sources.	Despite its targeted threat mitigation, it may fail to notice complex traffic patterns, leading to unnecessary rerouting and possible disruptions to benign traffic.
	Renyi Entropy (Ahalawat et al.^16^)	analyzed Renyi entropy for detection of DDoS by leveraging probability distributions in traffic analysis of SDN.	However, this technique potentially leads to false negatives or requires resource-intensive learning processes as it may struggle to differentiate DDoS attack traffic from high-volume legitimate traffic.
	CC-Guard (Wang et al.^17^)	proposed a technique named CC-Guard for managing workload distribution by emphasizing cross-domain routing and switch migration.	However, it under utilizes data available in flow tables, leading to generalized mitigation actions across all switches.
	SD-Anti-DDoS (Cui et al.^18^)	designed a comprehensive ‘SD-Anti-DDoS’ system incorporating attack detection, traceback, and mitigation phases. It utilizes the programmability feature of SDN to isolate and block malicious traffic dynamically. SD-Anti-DDoS significantly reduces SDN	But, its dependence on tracing the complete attack path for DDoS attack identification can lead to delays in activating the mitigation phase, particularly during high-volume or sophisticated DDoS attacks.
ML/DL Approaches	Entropy-NN Hybrid (Zhang et al.^19^)	combined entropy with triggered-based activation of neural networks and machine learning classifiers to reduce training time and computational demands.	However, their evaluation was based solely on dataset testing without validating real-time traffic in SDN. In addition, the use of fixed thresholds in entropy calculation may limit its broader applicability.
	OpenFlowSIA (Phan et al.^20^)	developed ‘OpenFlowSIA’, which adjusts flow idle timeouts in data plane switches and uses Support vector machine (SVM) classification outcomes to reduce resource exhaustion.	Despite its suitable implementation approach, this mechanism risks over-blocking legitimate traffic, if the SVM misclassifies it as DDoS traffic.

To summarize, the existing research work often presents a trade-off among solutions that are either fast but inadaptive (statistical methods), accurate but resource-intensive (ML methods), or efficient but coarse-grained (non-targeted mitigation). To overcome these limitations, this research proposes a three-tier defence mechanism that is lightweight, adaptive, and event-driven. This defence mechanism exploits real-time flow-table data, detects unusual traffic quickly, and applies targeted mitigation measures by focusing on attack sources and affected nodes, without additional controller overload or unnecessary traffic blocking. The proposed solution incorporates three tiers of protection: an improved lightweight adaptive Cumulative Sum (CUSUM) algorithm for swift DDoS detection at the first tier, and an event-driven machine learning model employing a Decision Tree (DT) classifier at the second tier. The second layer is only activated when the first tier identifies a potential vulnerability against the DDoS. In this way, it further confirms the existence of DDoS in cases of false alarms (by tier I) and drastically reduces the controller’s computational load and false positive rates. In the final tier, a targeted mitigation technique known as port connection analysis is incorporated to precisely identify the direct source port connected to the host that initiated the attack. By centralizing the focus on the source without tracing the complete attack path of malicious traffic, the proposed mechanism provides a more nuanced and adaptive response to evolving DDoS attack trends.

The main contributions of this research paper are as follows: 1) DDoS detection with minimal Latency: The proposed CUSUM algorithm of the first-tier approach detects traffic anomalies within one second, with a highly reduced latency for DDoS detection and swift response to the potential threat. 2) An event-based activated verification tier: The second layer applies a DT classifier activated only after receiving an anomaly detection trigger from the first tier. It verifies attacks with 99.99% accuracy, minimises the controller’s computational load, and reduces false positives by 87% compared to standard methods. 3) An efficient and targeted mitigation mechanism: In the third tier, it performs an in-depth analysis of the ports connecting to show direct and indirect port connections to the attack sources by utilising the link-layer discovery protocol of the Ryu controller, reducing non-actionable mitigation by 94% while preserving network performance through effective neutralisation. 4) Scalable defence framework: The proposed mechanism has been fully experimented with via simulation across a variety of network topologies, demonstrating its capability to maintain operation with zero packet loss for legitimate traffic, with minimal impact on overall network performance.

The subsequent sections of this work are organized as follows: The following section discusses the testing approach of the proposed method. The third section covers the detailed methodology of each proposed tier of defense mechanism. The result and discussion section discuss the performance outcomes of the proposed research work. The last section concludes the work and outlines future research directions.

## Three-tier defense mechanism experimental setup and pre-test results

This section covers the approach used to conduct experiments in this research. The methodology employed in this study is structured into several key components. Among these, selecting the most appropriate ML classifier and feature selection is a foundational step, as they directly impact the performance of the detection system in SDN. Table 4 lists the hardware and software configuration tools used for the proposed DDoS defence mechanism in SDN environments.

**Table 4:** List of hardware and software configuration used for proposed DDoS defense mechanism in SDN environments.

**Category**	**Component/Tool**	**Description/Purpose**
Hardware & Software	Computer	AMD Ryzen 7 5800H, 8 GB RAM
	Operating System	Dual-boot: Windows 11 & Ubuntu 20
	Mininet Network Simulator	Simulate SDN environment
	Ryu Controller	Manage simulated SDN network
	Topology Setup	Custom topologies for experiment comparison
	D-ITG, IPERF, PING	Generate normal network traffic
	Hping3	Replicate DDoS attacks (synflood, udpflood, icmpflood)
	htop	Monitor average CPU utilization (%)
	Python	Build three-tier defense mechanism
	Jupyter (Anaconda3)	Integrated development environment
	Scikit-Learn, psutil, joblib etc	ML, monitoring, and parallel processing libraries

### Classifier selection based on SDN-specific datasets

A classifier plays a significant role in the development of ML-based defense mechanisms against DDoS attacks. It is also essential to use the datasets that reflect the unique traffic patterns of SDNs for assessing these classifiers. In existing works, classifiers are analysed either based solely on datasets generated for traditional network traffic patterns or using highly biased datasets (e.g., CICDDoS2019 and InSDN), as they contain mostly (99.99%) DDoS traffic. Therefore, in this study, we tested the classifier trained on both datasets, which were designed to reflect benign and malicious traffic patterns within SDN. These SDN-specific datasets offer a more accurate context for evaluating classifier performance in SDN than those datasets generated for traditional networks. The classifiers examined in this study include AdaBoost, Random Forest, Decision Tree, k-Nearest Neighbors (KNN), and Support Vector Machine (SVM) classifier.

We downloaded the first dataset from IEEE Dataport^21^. It has 103,346 traffic instances spread across 25 features. The authors have generated this dataset using the Mininet emulator to model the challenges faced in cloud computing and SDN environments. However, the authors of dataset2 (available on Mendeley^22^) also generated it using the Mininet emulator with ten different network topologies, capturing 23 features to represent a wide range of traffic attributes, all controlled by a single Ryu controller. The performance of five classifiers were evaluated using these datasets based on key metrics such as training time, prediction time, memory usage, and detection accuracy. Table 5 presents the performance analysis of the five selected classifiers. The results highlighted the DT classifier as the most effective classifier due to its fast processing capabilities (prompt training and prediction times) along with minimal memory requirements and high accuracy levels (above 99%) on both Datasets.

**Table 5:** Performance comparison of different classifiers on dataset1 and dataset2.

**Classifier**	**Metric**	**Dataset1/Dataset2**
Random Forest	Training Time (s)	5.97/6.45
	Prediction Time (s)	0.16/0.18
	Memory Usage (MB)	18/16
	Accuracy (%)	99.99/100
Decision Tree	Training Time (s)	0.44/0.41
	Prediction Time (s)	0.015/0.0
	Memory Usage (MB)	18/16
	Accuracy (%)	99.98/99.99
AdaBoost	Training Time (s)	4.04/3.77
	Prediction Time (s)	0.20/0.18
	Memory Usage (MB)	22/20
	Accuracy (%)	99.96/99.54
KNN	Training Time (s)	0.02/0.004
	Prediction Time (s)	51.49/57.24
	Memory Usage (MB)	15/14
	Accuracy (%)	88.74/88.52
SVM	Training Time (s)	333.19/254.08
	Prediction Time (s)	140.9/109.92
	Memory Usage (MB)	22/25
	Accuracy (%)	67.6/67.4

### Evaluation of feature selection methods

The next step after selecting a classifier is to fine-tune the detection model by choosing the most relevant features for near-real-time detection in SDN. This step is known as the feature selection method. Feature selection helps us in identifying the most critical features that contribute the most to system detection accuracy. By selecting only relevant features, the dimensionality of the data is reduced, which ultimately decreases the computational load of the SDN controller.

**Fig. 1:**
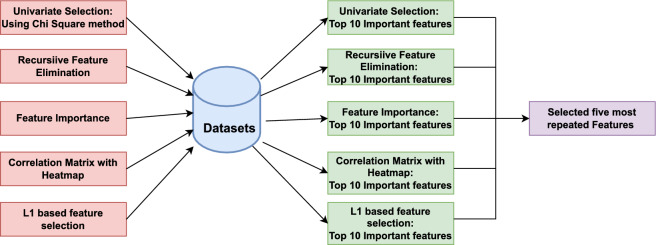
Methods used for feature selection on SDN-specific datasets.

In this work, we have applied various advanced statistical and machine learning-based feature selection techniques to identify the features that most accurately classify traffic. We employed five feature selection methods on two selected datasets (discussed in the previous subsection), namely, univariate selection, also known as the chi-squared method^23^, recursive feature elimination (RFE)^23^; the feature importance method^24^; the correlation matrix with the heatmap method^23^ and the L1-based feature selection method by utilizing Lasso regression^24^. The methods used to finalize the feature selection for this methodology are shown in Fig. 1. We created a structured approach to consolidate the findings and identify the most frequently chosen features across both datasets after implementing these methods. And, extracted the top 10 most prominent features that played the most significant role in accurately detecting DDoS attacks using each technique. The result of this process was to identify the top five features that these feature selection methods consistently selected across all datasets.

At last, the DT classifier’s performance was again evaluated using three different feature sets: the complete feature set of the datasets, the top 10 most frequently occurring features, and the top 5 most frequently occurring features across both datasets, as shown in the Tables 6 and 7. The purpose of this test was to analyse the impact of feature selection on the overall performance of the classifier. The results are illustrated in the Table 8 proves that reducing the number of features does not impact the accuracy of the defence mechanism. The effectiveness of the proposed Tier I and II was further quantified utilizing both datasets and simulated real-time network traffic through a comprehensive comparative analysis. The findings presented in Table 9 highlight the superior accuracy of the proposed Two-tier approach on SDN-specific datasets, its validation accuracy (%), and its Detection Rate (DR), surpassing the benchmarks established by current methodologies.

**Table 6:** Top ten most frequently selected features from dataset1 and dataset2.

**Feature**	**Frequency (Dataset1)**	**Frequency (Dataset2)**
pktcount	5	5
bytecount	5	5
pktperflow	4	5
byteperflow	4	4
pktrate	4	4
dur_nsec	4	3
dt	4	3
tot-dur	3	-
src	3	-
rx_bytes	3	-
packetins	-	3
pairflow	-	2
switch	-	2

**Table 7:** Top five most frequently selected features from both datasets.

**Feature**	**Frequency (Dataset1)**	**Frequency (Dataset2)**
pktcount	5	5
bytecount	5	5
pktperflow	4	5
byteperflow	4	4
pktrate	4	4

**Table 8:** Accuracy of decision tree classifier across different sets of features.

**Features**	**Accuracy on Dataset1**	**Accuracy on Dataset2**
All features	99.99%	99.99%
Top 10 selected features	99.97%	100%
Top 5 selected features	99.95%	99.98%

**Table 9:** Performance comparison of proposed first two tier based on utilized datasets with existing mechanisms for detection of DDoS in SDN.

**Ref**	**Dataset**	**Classifier**	**Accuracy (%)**	**Validated Accuracy (%)**	**DR**
**[29]**	Dataset1	NB	99.91	-	1
		NB-Multinimial	99.91	-	1
		RBFC	99.91	-	1
		RBFN	99.91	-	1
		LR	99.91	-	1
		SLR	99.91	-	1
		SMO	99.91	-	1
		BN	99.46	-	0.9
		J48	99.91	-	1
		NB-Tree	99.46	-	0.9
		SWAST-HIK	99.46	-	1
**Proposed (Tier I and II)**	**Dataset1**	**Decision Tree**	**99.95**	**99.99**	**1**
**[30]**	Dataset2	CNN	98.74	-	0.9
		SVC-SOM	95.45	-	0.9
		SAE-MLP	99.75	-	0.9
		CNN-LSTM	99.48	-	0.9
**[31]**	Dataset2	LSTM	92.6	76.43	0.8
		SVM	-	95	0.9
		XGBoost	99.55	99.54	-
		CNN-LSTM-AE	99.99	99.99	1
**Proposed (Tier I and II)**	**Dataset2**	**Decision Tree**	**99.98**	**99.99**	**1**

### Switch response with and without port connection analysis

This study examined the impact of mitigation techniques on the integrity of normal traffic under DDoS attack conditions across three distinct SDN topologies: Single, Linear, and Tree. Each of these topologies was assessed for how well the network could maintain the delivery of legitimate packets with and without the implementation of port connection analysis during an active DDoS attack. Figures 3(a, d and g) provide a graphical representation of the topologies used, showing the direction of both DDoS attack traffic: malicious data packets intended to overwhelm the network and normal traffic in single, linear, and tree arrangements.

**Fig. 2:**
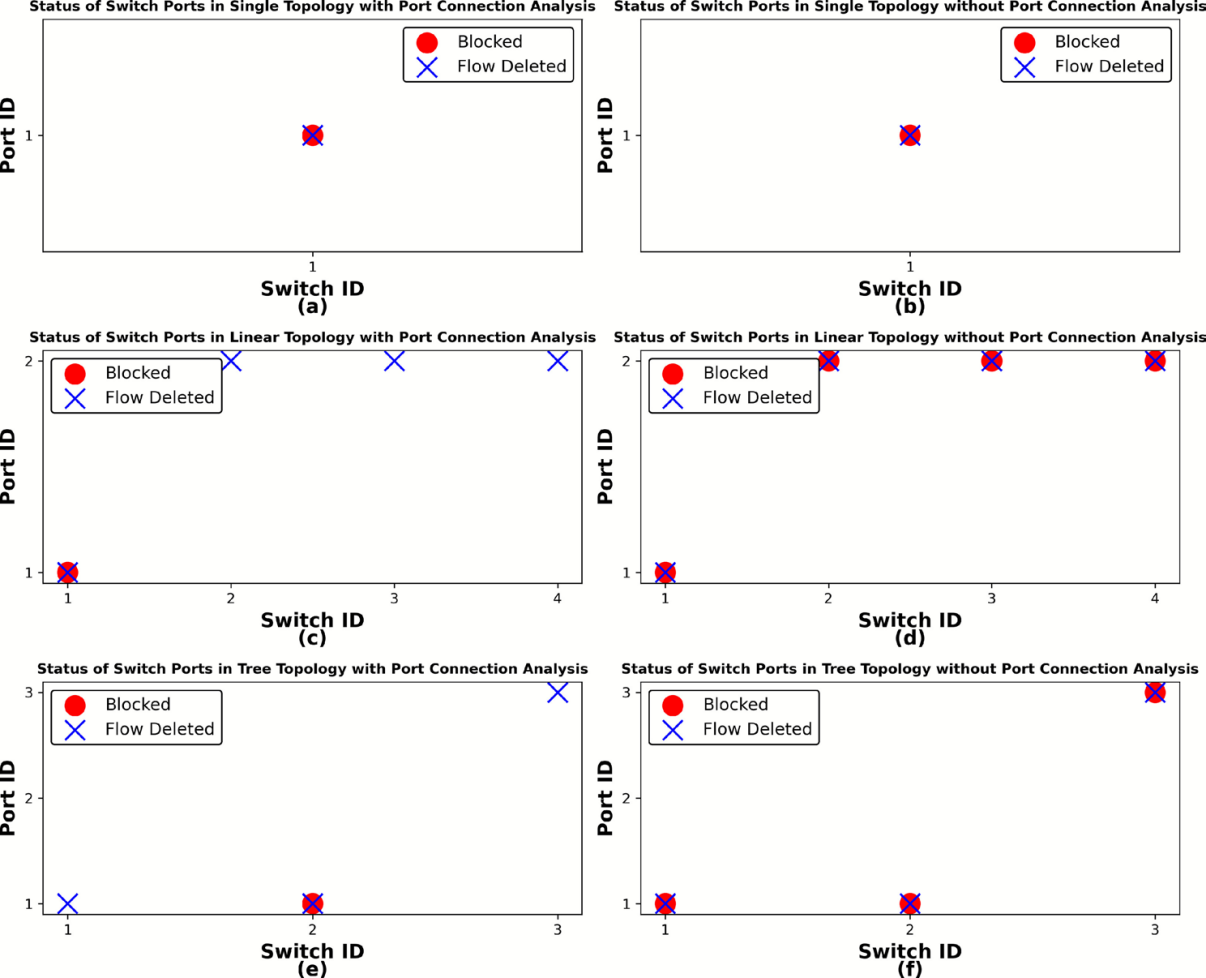
Comparison based on switch ports status with and without port connection analysis method under DDoS attacks condition across three distinct SDN topologies.

**Fig. 3:**
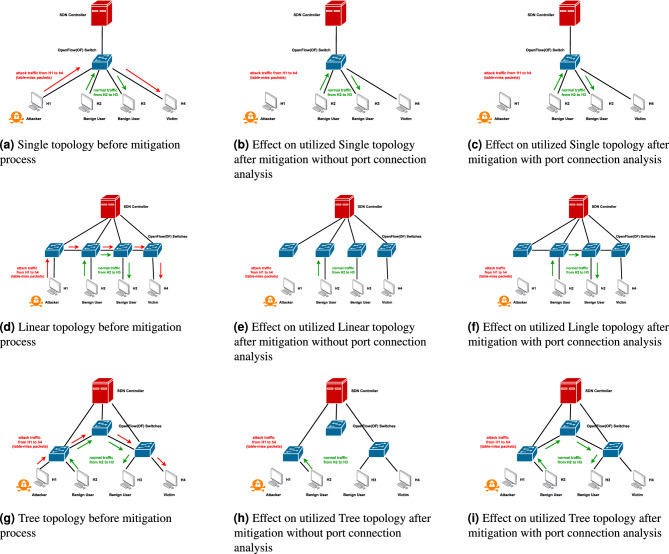
Graphical representation of impact of mitigation mechanism (with or without port connection analysis) on different SDN network topologies.

The Hping3 tool, a network packet generator, was used to create simulated DDoS traffic, while the ’ping’ command generated standard, low-frequency network requests between their respective hosts. During these tests, we maintained the same traffic patterns in all the three topologies: H1 sent simulated DDoS attack traffic to H4, while H2 sent legitimate (normal) traffic to H3. This configuration was designed to test whether the mitigation technique could successfully distinguish between malicious and legitimate traffic while maintaining uninterrupted service for normal traffic. The results indicated that the system using port connection analysis applied both blocking (preventing communication) and flow deletion (removing forwarding rules for malicious traffic) only to the attack entry switch. The remaining victim switches observed only flow deletion, meaning that only suspicious traffic rules were removed based on the attack origin and flow path. In contrast, when port connection analysis is not used, all switches universally apply both blocking and flow deletion, which can potentially cause more network disruption. This difference is illustrated in Fig. 2(a to f). Figures 3(b-c, e-f, and h-i) graphically demonstrate that the mitigation strategy with port connection analysis prevents disruption to regular network traffic; the approach maintains efficient legitimate traffic flow and affirms its non-disruptive nature.

## Research Methodology

**Fig. 4:**
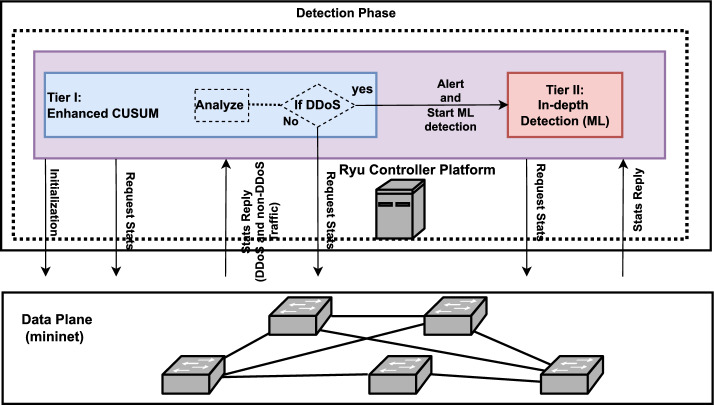
Architectural diagram of proposed first two-tiers (detection phase) of defense mechanism.

**Fig. 5:**
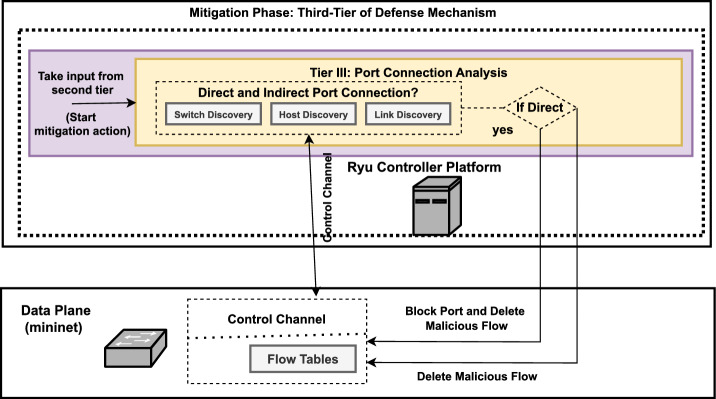
Architectural diagram of proposed third tier (mitigation phase) of defense mechanism.

To detect and respond to potential DDoS threats, a novel three-tier defense mechanism is implemented within the control plane of SDN. This mechanism is divided into two main phases: detection and mitigation. The detection phase utilizes flow statistics from data plane and consist of first two tiers. An enhanced CUSUM algorithm at the first tier, and an trigger activation based ML model using a DT classifier in the second tier. The third tier at the mitigation phase employed the novel port connection analysis technique to block and remove identified DDoS attack traffic at the source port without tracing the complete attack path. The proposed method aims to balance rapid detection with efficient and speedy mitigation response by combining statistical and machine learning techniques at the control plane of SDN. The Figs. 4 and 5 illustrate the architectural diagrams of proposed detection and mitigation phase of three-tier defense mechanism.

### Tier I: enhanced CUSUM algorithm for DDoS attack detection

In the first tier, CUSUM acts as an effective statistical tool for spotting sudden changes in traffic which may indicate a DDoS attack. However, it relies heavily on setting the right parameters, such as thresholds and window sizes. Finding the optimal configuration for different network conditions can be difficult. CUSUM is highly sensitive to sudden changes and therefore, it may wrongly flag harmless traffic spikes even in the event of benign flash traffic as potential DDoS attacks. Prolonged normal activity might also surpass thresholds, causing false alarms. To address these issues, an enhanced version of the CUSUM algorithm is incorporated with additional features e.g., adaptive threshold, sliding window and multidimensionality of data, along with second tier of a DT classifier which is trained on historical data and activated when CUSUM detects unusual activity. The CUSUM algorithm implemented at the control plane of SDN, continuously monitors flow statistics collected from the SDN data plane (switches), such as packet and byte counts, and applies adaptive thresholds to detect any abnormality in network traffic. The following sections cover a detailed explanation of: the collection of flow statistics, adaptiveness of thresholds, management of data within a sliding window, and the utilization of multidimensionality in the process.

#### Collection of flow statistics

At regular intervals, flow statistics are collected by SDN controller from the switches (i.e., data plane of the SDN). This is achieved through the Ryu controller framework that sends out requests for flow statistics to each switch in the network. The Ryu controller periodically requests this data (flow statistics) from SDN switches. Upon receiving these statistics, the system updates its internal data structures with the latest packet and byte counts (denoted as $P_t^f$ and $B_t^f$) for every flow *f* at time *t*. A flow is defined by its match fields, including source and destination IP addresses, source and destination ports, and the input port of the switch. The algorithm’s subsequent calculations require these counts as a fundamental input.

#### Sliding window

To make the CUSUM algorithm capable of handling the most recent data, we incorporate an additional feature: a sliding window as a queue data structure. It is one of the key features of our proposed CUSUM detection algorithm. It enables the CUSUM algorithm to shift forward over time while maintaining its fixed size and containing the most recent data points, such as packet and byte counts. By doing so, it enables the analysis of the most recent flow statistics while discarding older data that may no longer be relevant for calculating the cumulative sum. As new data arrive se.g., $P_{t+1}^f$ and $B_{t+1}^f$, it is appended to the queue window, while the oldest observations are discarded without expanding the window size. This mechanism allows the real-time adjustment of calculated metrics and the thresholds.

#### Adaptive thresholds

For real-time changing network traffic models, a fixed threshold value has the following disadvantages: a) lack of scalability; b) unable to accommodate the changing trend of the network. Therefore, to tackle these issues, an adaptive threshold adjustment algorithm is applied, which calculates the mean and standard deviation of the sequence $X_i$ in real-time with a fixed-size sliding window (length L=50). Adaptive thresholds algorithm incorporated in proposed CUSUM algorithm is used to determine whether the observed traffic is legitimate or potentially malicious for a specific flow. The mean provides a baseline for expected legitimate or normal traffic, while the standard deviation accounts for regular variability in incoming network traffic. The thresholds are adjusted dynamically according to observed traffic characteristics, making the detection mechanism sensitive to sudden changes while accommodating normal fluctuations in network traffic. The proposed enhanced version of CUSUM algorithm determines whether an abnormality occurs in data sequence through the threshold value *T* in the decision function $D_T$, which expressed in equation 1:

1 $$\begin{aligned} D_T = {\left\{ \begin{array}{ll} 0, & \text {if } y < T \\ 1, & \text {if } y \ge T \end{array}\right. } \end{aligned}$$

Where *y* is the cumulative sum coefficient. The value of threshold changes when observed metrics has a linear relationship with a coefficient of $\theta$ (coefficient $\theta$ is a scaling factor to adjust sensitivit). For instance, thresholds for packet counts $T_{P,t}^f$, and byte counts $T_{B,t}^f$ of the sequence $X_i$ can be dynamically adjusted based on the observed data’s mean and standard deviation within the sliding window are expressed as 2 and 3:

2 $$\begin{aligned} T_{P,t}^f = \mu _{P,t}^f + \theta \cdot \sigma _{P,t} \end{aligned}$$

3 $$\begin{aligned} T_{B,t}^f = \mu _{B,t}^f + \theta \cdot \sigma _{B,t} \end{aligned}$$

where as $\mu$ and $\sigma$ are the mean and standard deviation of packet counts and byte counts for flow *f* and they are calculated as using 4, 5, 6, and 7:

4 $$\begin{aligned} \mu _{P,t}^f = \frac{1}{n} \sum _{i = t - n + 1}^{t} P_i^f \end{aligned}$$

5 $$\begin{aligned} \mu _{B,t}^f = \frac{1}{n} \sum _{i = t - n + 1}^{t} B_i^f \end{aligned}$$

6 $$\begin{aligned} \sigma _{P,t} = \sqrt{\frac{1}{n - 1} \sum _{i = t - n + 1}^{t} \left( P_i^f - \mu _{P,t}^f\right) ^2} \end{aligned}$$

7 $$\begin{aligned} \sigma _{B,t} = \sqrt{\frac{1}{n - 1} \sum _{i = t - n + 1}^{t} \left( B_i^f - \mu _{B,t}^f\right) ^2} \end{aligned}$$

#### Multidimensionality of features

Due to different types of attacks, the changes in network traffic are not the same. Moreover, features in a single dimension are not sufficient to fully capture changes in network traffic. In other words, feature selections in a single dimension will cause a very high missed detections rate. Therefore, in this research, the CUSUM algorithm will support multi-dimensional feature sequences for detection. Assume that the features are extracted in three dimensions. For each sliding window with their each respected flow f at time t, a decision function is constructed to represent a sequence $X_(i,1),X_(i,2)$, to obtain cumulative sum coefficients: $y_1,y_2$. Now the decision function of the kth dimension feature will become:

8 $$\begin{aligned} D_{T_k} = {\left\{ \begin{array}{ll} 0, & \text {if } y_k < T_k \\ 1, & \text {if } y_k \ge T_k \end{array}\right. } \end{aligned}$$

For each flow f at time *t*, a decision function $D_N$ is constructed to represent the state of the network traffic. This vector includes attributes, such as: packet count $P_t^f$, and byte count $B_t^f$. Thus, a multi-dimensional decision vector will become, $D =(d_{N_1 },d_{N_2})$ and the final decision function is obtained as:

9 $$\begin{aligned} r(D) = {\left\{ \begin{array}{ll} 0, & \text {if } \Vert D\Vert < 1 \\ 1, & \text {if } \Vert D\Vert \ge 1 \end{array}\right. } \end{aligned}$$

**Fig. 6:**
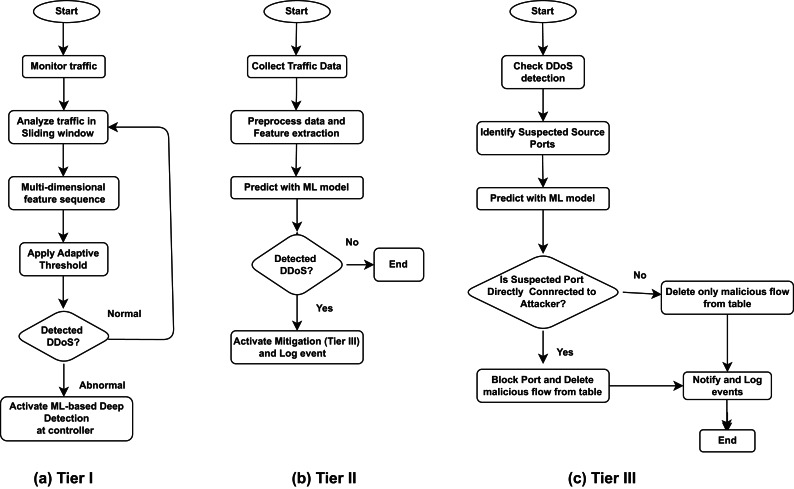
The step-by-step workflow of proposed (**a**) Tier I, (**b**) Tier II and (**c**) Tier III of defense mechanism.

This decision function in above equation implies that if the decision of any dimension feature is abnormal, the final decision result also becomes abnormal. If any component of D exceeds its corresponding threshold, the traffic is flagged for further analysis or immediate action, depending on the severity of the deviation. The Fig. 6(a) summarizes the algorithm flow of the improved CUSUM algorithm. This algorithm will be applied as the trigger detection module at first tier while the equation 4 to 7 shows the list of statistical features that are extracted and calculated to coarsely represent network traffic changes.

### Tier II: verification of DDoS attack using DT classifier

The implementation of DT classifier in the second tier of detection phase improves accuracy by distinguishing genuine DDoS attacks from legitimate network traffic. When CUSUM flags suspicious traffic and activate second tier classifier, the DT classifier further confirms whether it is a DDoS or legitimate traffic. The system activates the DT classifier only after receiving an alert from CUSUM. This event-based activation of the DT classifier ensures that machine learning analysis runs only when necessary. The DT classifier is trained on historical data to recognize complex patterns of DDoS traffic. This two-step approach helps in increasing accuracy, reducing false positives rate and computational load of SDN controller, while ensures that only actual DDoS attacks lead to third tier i.e., mitigation phase.

Figure 6(b) illustrates a step-by-step procedure of a DT classifier at the second tier of the detection phase for identifying DDoS attacks traffic within network traffic. It describes the sequence of feature extraction, machine learning-based prediction, and the subsequent decision-making process based on the classifier’s output.

### Tier III: targated mitigation with port connection analysis

Before detailing the proposed third-tier mitigation methodology, it is essential to understand the basic network structure of SDN, as the switches at the data plane of SDN are relatively simple entities compared to those in traditional networks. It only keeps the record of traffic flow from source to destination. When the DDoS attacker floods the SDN with fake requests, these requests are stored not only on the source switch where the attacker initiated the attack, but also on the intermediate switches that these requests take to reach the controller or the destination. Therefore, all these switches (source and those that come in the traffic path) are overloaded with fake requests, and they all raise the flag for malicious activities, as there is no strategy applied at the detection phase to distinguish the source switch from intermediate switches. Therefore, when mitigation is employed by analyzing only corrupted ports without implementing a targeted strategy, it will eventually block all switches that are part of an attack traffic path and disrupt the overall network service.

An alternative approach is to track the complete path of attack requests. Since the request comes in bulk and tracing the full path would take a significant amount of time, the network may already be compromised by a DDoS attack. Therefore, we need a more targeted mitigation strategy. Once the controller receives the alert of switch corruption, it immediately checks its connection with the attacker host (whether it is connected directly or through intermediate nodes or switches). This swift mapping will enable a faster response to DDoS attacks without compromising the network’s security for an extended period. In this work, we address these limitations by providing a more targeted and effective mitigation response at the third tier for mitigation of DDoS at the control plane, rather than the data plane of SDN. The recommended targeted mitigation aims to ensure a balanced defence against DDoS attacks while preserving service availability and minimizing collateral damage.

The proposed third tier at the mitigation phase utilizes network topology discovery technologies to map the network’s structure and identify the connections between hosts (endpoints) to switches at the SDN data plane. It provides context to distinguish between direct and indirect port connections. The system constantly gathers information about switches, hosts, and their connections by tracking Link-Layer Discovery Protocol (LLDP) messages and processing host attachment events (such as which host is connected to which switch).

The suggested system continuously generates a dynamic map of the network topology, showing which devices (hosts) are connected to switches via specific ports and the associated traffic routes. Whenever it detects a possible DDoS attack, the system examines the topology map to determine how the suspected source (port) connects to the switch (e.g., host to switch/switch to switch). We consider it a direct port connection if the host is connected to the compromised switch. An indirect port connection scenario occurs when traffic is received through a different data plane switch. This analysis determines the appropriate mitigation strategy (such as port blocking and flow deletion or just flow deletion). If a direct connection is confirmed, the system blocks and deletes the flow table entries from the compromised switch port to prevent all traffic from the compromised port and prevent further disturbance. The system takes a different approach in the case of an indirect port connection. The purpose is to eliminate fraudulent flow entries related to DDoS, not to block the switch port, which could interfere with regular traffic. The technology safeguards against table overflow attacks and ensures valid flows by eliminating these entries. The entire workflow of the suggested third-tier defence mechanism is depicted in Fig. 6(c).

### Probabilistic modeling and computational complexity of detection mechanism

This section describes the probabilistic models used in the initial two detection phase tiers and provides a comprehensive analysis of their complexity. The probability P(D) is essential in this context, as D here represents the event of a DDoS attack being accurately identified by CUSUM. Whereas, $(1 - P)$ is the residual probability handled by the DT classifier. Ideally, the probability P(D) should be near or equal to one, indicating that the proposed improved version of the CUSUM algorithm at tier-I can identify DDoS traffic with complete accuracy. That ensures that only true positive DDoS traffic is forwarded to the subsequent processing stages (Tier II). Thereby optimising resource utilisation at the first tier. The computational complexity of each component is crucial for assessing efficiency and scalability within the detection system. These complexities are represented as *O*(*CUSUM*), which refers to the complexity associated with the first tier, and *O*(*DT*), which means the complexity related to the DT classifier used in the second tier of detection. The differential complexities of the CUSUM algorithm must be less than the DT classifier as referred in equation 10. Therefore, considering the probabilistic detection at the detection phase, the total complexity of the system can initially be expressed in equation 11:

10 $$\begin{aligned} O(\text {CUSUM}) \ll O(\text {DT}) \end{aligned}$$

11 $$\begin{aligned} O(\text {Total}) = O(\text {CUSUM}) + O(\text {DT}) \end{aligned}$$

The refined total complexity at the proposed detection phase is formulated as:

12 $$\begin{aligned} O(\text {Total}) = P(D) \cdot O(\text {CUSUM}) + (1 - P(D)) \cdot O(\text {DT}) \end{aligned}$$

Above equation 12 dynamically adjusts the computational load based on the accuracy of the CUSUM algorithm. In an ideal scenario where $P(D) = 1$ (representing perfect accuracy and no false negatives), the total complexity simplifies, as shown in equation 13:

13 $$\begin{aligned} O(\text {Total}) \cong O(\text {CUSUM}) \end{aligned}$$

Thus, the system’s efficiency is significantly enhanced when the CUSUM algorithm verifies DDoS traffic, bypassing the computationally heavier ML model.

## Results and discussion of proposed three-tier defense mechanism

### Evaluation of the performance of the proposed two-tier detection mechanism under different intensities of DDOS attack and benign flash traffic

**Fig. 7:**
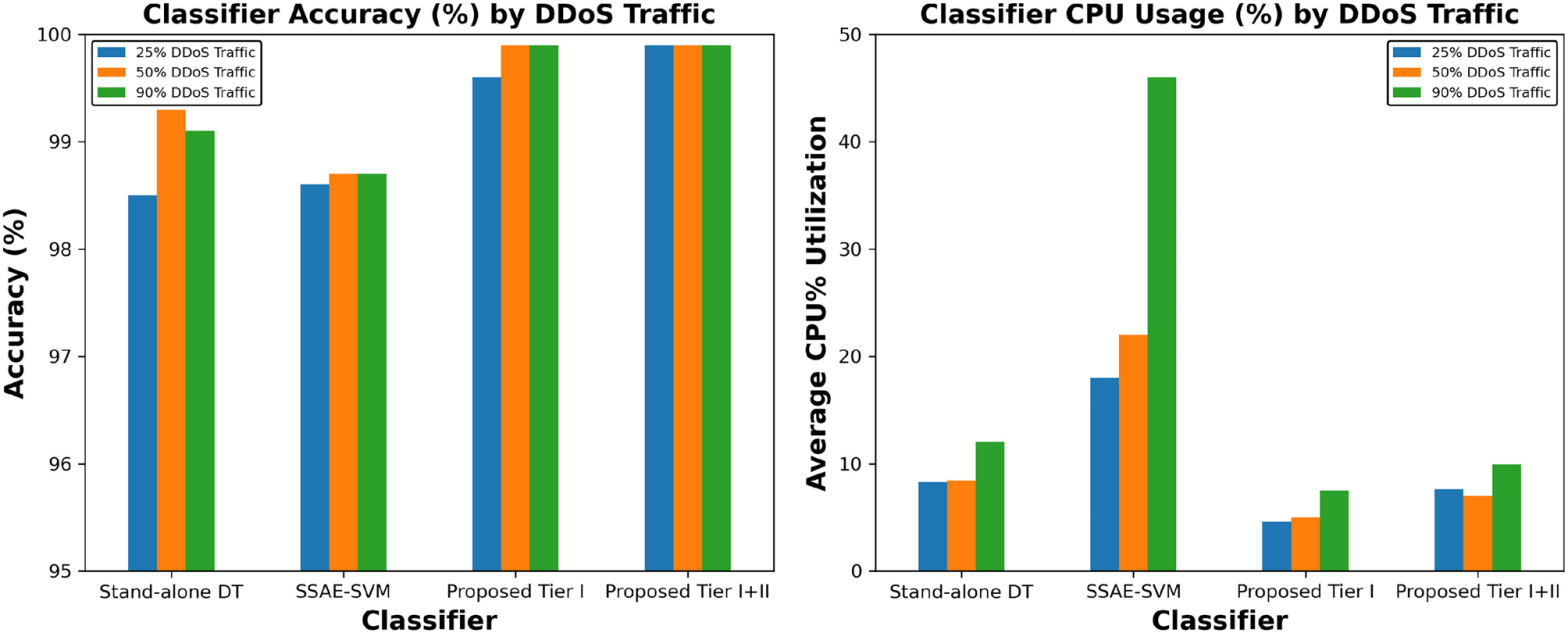
Analysing the performance of proposed Tier I, Tier I+II mechanism, Standalone DT classifeir and SSAE-SVM accuracy and average CPU utilization over different intensity of DDoS attack.

Accurately differentiating between malicious and benign traffic is crucial for DDoS defence mechanisms. This study explored this area by implementing a two-tier detection mechanism designed to distinguish between sudden high-volume traffic generated by DDoS attacks and benign traffic. The first experiment was conducted to evaluate the accuracy of the proposed tier I and proposed tier I+II with a standalone DT classifier and the existing SSAE-SVM^19^ work. We tested the system under various intensities of DDoS traffic. For this study, we utilise an identical network topology simulated by Mininet, which includes one controller and one switch with 20 hosts, to assess a fair comparison among the proposed mechanism, the standalone DT classifier, and the SSAE-SVM^19^ techniques. All the mechanisms were fairly tested with three different intensities of DDoS attack: 1) 25% DDoS traffic + 75% normal traffic, 1) 50% DDoS traffic + 50% normal traffic, and 1) 90% DDoS traffic + 10% normal traffic. Figure 7 (left) concludes that the proposed two-tier detection approach performs better compared to the standalone DT classifier and SSAE-SVM^19^ in all three intensities of DDoS attacks. However, the performance of the proposed tier I is slightly improved due to the second tier, particularly when 25% of the generated traffic was DDoS. While Fig. 7 (right) compares defence mechanisms based on their average CPU (%) utilisation; hence, the proposed tier I+II proves not only to be accurate but also a lightweight defence mechanism compared to the standalone DT classifier and SSAE-SVM.

**Fig. 8:**
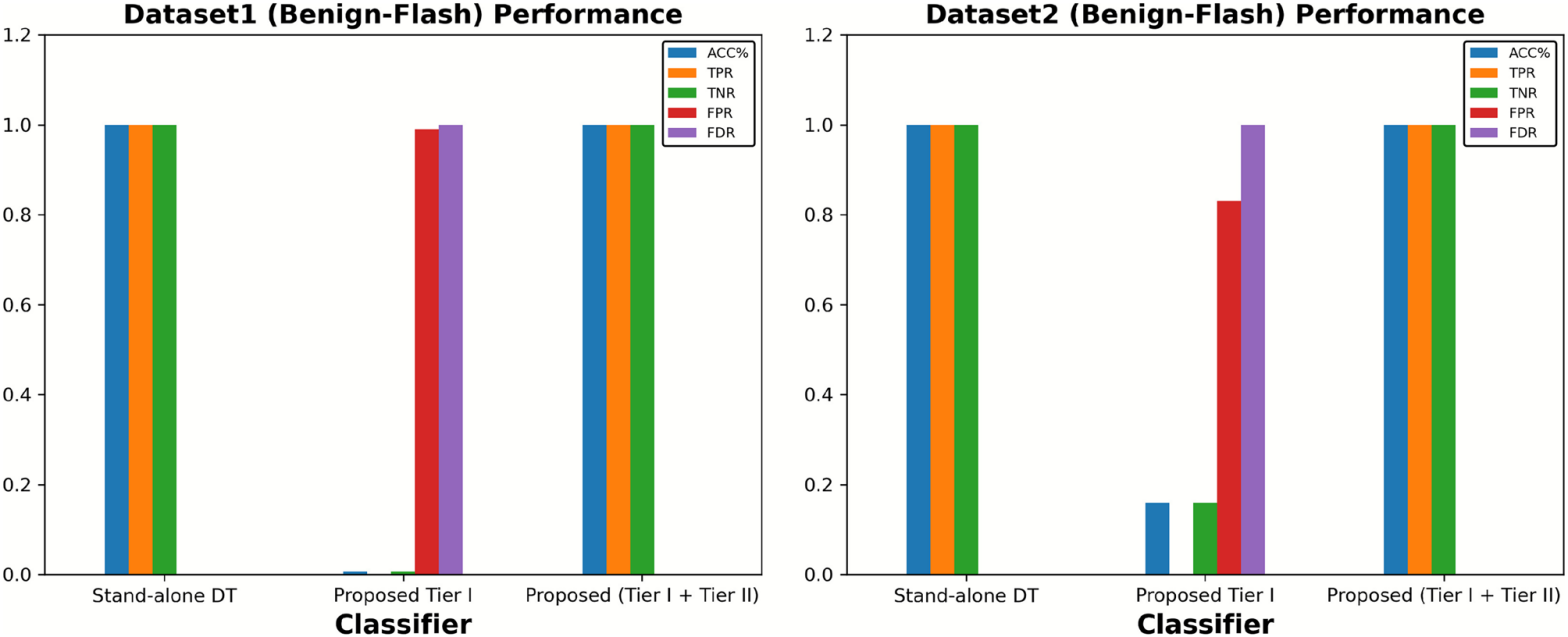
Performance comparison among proposed Tier I, Tier I+Tier II and Stand-alone DT in context of benign flash traffic.

Now, the question arises as to why we need a second ML-based tier when the proposed improvement to the CUSUM-based defence mechanism at tier I performs as accurately and is also lightweight compared to the proposed tier I+II? As we know, the CUSUM algorithm works by detecting sudden high-volume changes in network traffic by comparing it with a normal network traffic threshold. What happens if the sudden increase in volume is caused by normal traffic, rather than DDoS flooding of traffic? Will the proposed Tier I still be able to distinguish itself as normal despite the similar volumetric increase in traffic? To test this scenario, we evaluate the performance of the proposed tier I detection model with benign flash traffic. To achieve this, we carefully designed a network simulation to represent a real-world reality situation, interspersed with periods of high and realistic traffic. A single topology with a single Ryu controller and four switches, each attached to a single host. The characteristics of the flash crowd event (a benign surge in network traffic) were replicated using iperf in Mininet. Three out of four hosts (h2, h3, and h4) were set up in this simulated environment to send a flood of data packets in the direction of a target host (h1), each at a rate of 1000 packets per second. Figure 8 (left) clearly demonstrates that the proposed first tier, while lightweight and CPU-efficient, struggled to distinguish a sudden increase in traffic caused by DDoS or benign traffic. That resulted in poor Tier I accuracy and high false positive rates when exposed to benign flash traffic scenarios. The incorporation of an ML-based second tier, activated only upon specific network events, marks a significant enhancement in detection capabilities. We then tested the hybrid approach against benign flash events. The results are reflected in Fig. 8 (right), clearly demonstrate the need for the second tier in the event of sudden surges in normal network traffic.

### Effect of the implemented mitigation mechanism on different SDN topologies

**Fig. 9:**
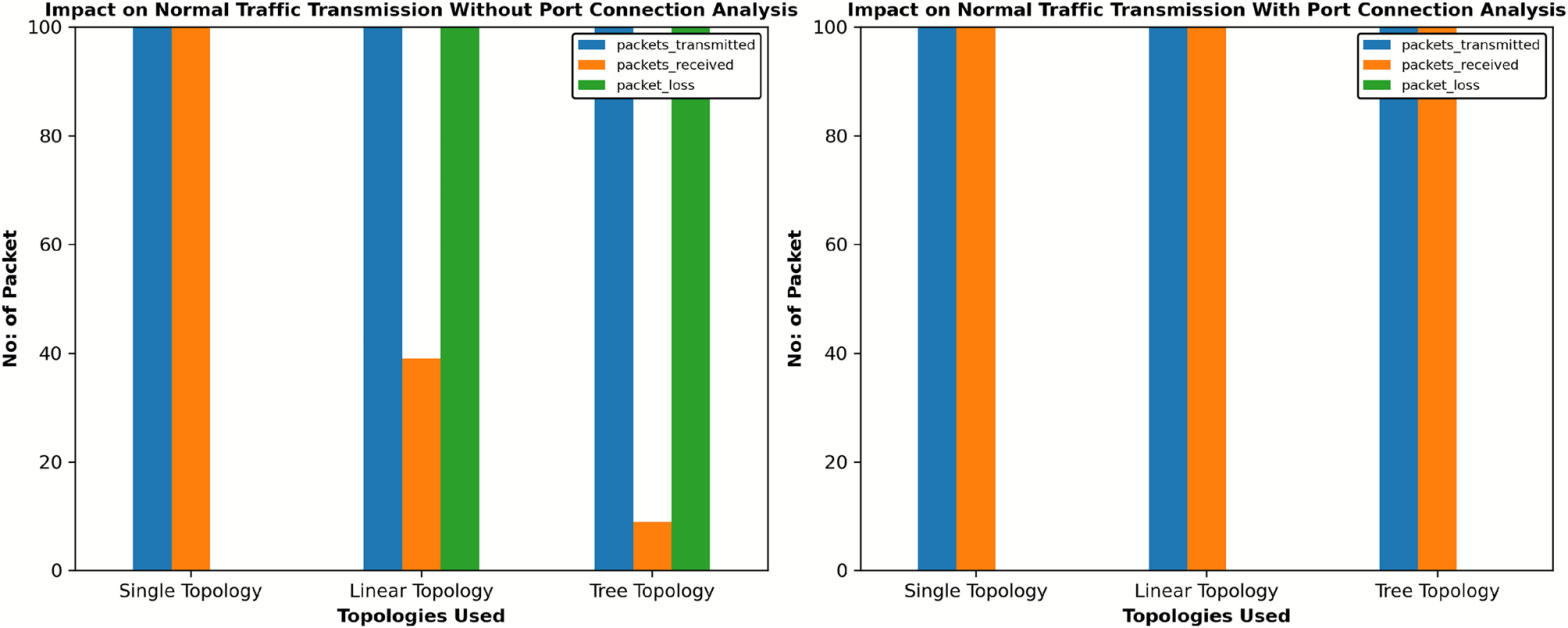
Comparison based on impact on normal traffic transmission with proposed port connection analysis mitigation technique.

We examine how different network topologies were affected by targeted mitigation techniques during DDoS attack conditions. We tested our proposed port connection analysis method with three different topologies and compared it with non-targeted mitigation work. The findings are shown in the Fig. 9 emphasise the importance of targeted mitigation strategies with LLDP for detailed port connection analysis to protect legitimate network operations during DDoS attacks. We observe that in a single topology, our proposed mitigation techniques completely prevent packet loss, proving the method’s effectiveness in distinguishing between legitimate and malicious traffic without disrupting normal traffic flow. On the other hand, the same topology suffered a significant 41% packet loss, implying that general mitigation approaches can unintentionally disrupt normal traffic flow of the network. Similar 0% packet loss was observed in both linear and Tree topologies, with the applied port connection analysis proving its efficiency across longer network paths. However, without targeted mitigation, packet loss increased to 74% in linear structures and 94% in tree topology. These results demonstrate that, without targeted analysis, network topology is the most severely affected, exposing its vulnerability to widespread blocking that can disrupt multiple branches of network traffic.

### Performance comparison between port connection analysis and attack trace mitigation methods

In this subsection, we have evaluated the performance of the proposed port connection analysis method against the existing attack path trace method, SD-Anti-DoS^18^. In order to ensure a fair comparison between the two techniques, we have tested our proposed method with an identical network topology that includes 25 switches and 200 hosts with a similar simulated traffic load. The SD-Anti-DoS technique examines the entire attack routes in order to analyzes all source ports and subsequently blocks relevant ports while deleting malicious traffic from only ports that fall within the path of attack traffic. This comprehensive tracing requires extensive coverage of attack vectors, resulting in delay in response times after detection of DDoS attacks. Table 10 shows that SD-Anti-DDoS^18^ requires roughly 15 seconds to initiate mitigation after attack detection. In contrast, our proposed port connection analysis method provides a swift response by scanning the connections of suspicious ports using LLDP, regardless of whether the ports are connected to an attack host or used as middle ports connected to switches in their attack path. The obtained findings demonstrate that our proposed method implements mitigation measures in approximately 0.002 seconds after detecting a DDoS attack, without disrupting the normal flow.

**Table 10:** Comparison on basis of response time taken by proposed port connection analysis mitigation method with benchmark method (SD-Anti-DDoS)

Method	Attack detection time (seconds)	Time to implement mitigation (seconds)
SD-Anti-DDoS [22]	$\sim$1	$\sim$15
Proposed Port Connection Analysis	$\sim$1	$\sim$0.002

## Conclusion and future works

A generic model approach for detecting and mitigating DDoS attacks in SDN has been presented in this research work. The work represents the effects of integrating statistical and ML approaches for detection and utilizing LLDP for the proposed port connection analysis method during mitigation, all on a single SDN controller. In essence, the following findings were observed at the detection phase. (1) The improved CUSUM algorithm, implemented at the first tier, uses features like a sliding window, an adaptive threshold, and multidimensional data analysis. This offers a faster, adaptive, and accurate detection model for SDN. (2) The improved CUSUM algorithm, while accurate, is still unable to distinguish between sudden benign flash traffic and attacks. It can trigger unnecessary mitigation actions. (3) The event-based activation of the DT classifier at the second tier overcomes the CUSUM limitation. It verifies the presence of DDoS attacks without continuously testing all incoming traffic data. The results show that the first two tiers of detection not only improve detection accuracy to 99.99% with minimal average CPU usage, but also decrease false positives by 87%. This guarantees more accurate mitigation actions. The mitigation phase at the final layer identifies both direct and indirect source ports without blocking switches that are not directly involved in the attack. This results in a 94% decrease in unnecessary mitigation actions while preserving network service continuity. The proposed strategy demonstrates substantial benefits over existing methods, particularly in high-traffic and complex attack scenarios. The mechanism achieved a response time of 0.002 seconds after detecting threats, compared to the 15 seconds needed by conventional methods like SD-Anti-DDoS with zero packet loss for legitimate traffic. This highlights its efficacy and minimal impact on network management. The quantitative findings emphasize the robustness and scalability of the proposed framework, effectively safeguarding against future DDoS attacks while preserving network performance. In the future, we will investigate the suitability of our proposed techniques for hybrid, multi-controller, and cloud systems, with the aim of developing a more flexible, self-learning system that continuously refines its detection algorithms in response to new attack patterns.

## Data Availability

The datasets used during this research work are cited in the article.

## References

[1] Kreutz, D. et al. Software-defined networking: A comprehensive survey. *arXiv preprint* arXiv:1406.0440 (2014).

[2] Scott-Hayward, S., O’Callaghan, G. & Sezer, S. Sdn security: A survey. In *2013 IEEE SDN For Future Networks and Services (SDN4FNS)*, 1–7 (IEEE, 2013).

[3] Shin, S., Yegneswaran, V., Porras, P. & Gu, G. Avant-guard: Scalable and vigilant switch flow management in software-defined networks. In *Proceedings of the 2013 ACM SIGSAC conference on Computer & communications security*, 413–424 (2013).

[4] Wang, H., Xu, L. & Gu, G. Floodguard: A dos attack prevention extension in software-defined networks. In *2015 45th Annual IEEE/IFIP International Conference on Dependable Systems and Networks*, 239–250 (IEEE, 2015).

[5] Piedrahita, A. F. M., Rueda, S., Mattos, D. M. & Duarte, O. C. M. Flowfence: a denial of service defense system for software defined networking. In *2015 Global Information Infrastructure and Networking Symposium (GIIS)*, 1–6 (IEEE, 2015).

[6] Dridi, L. & Zhani, M. F. A holistic approach to mitigating dos attacks in sdn networks. *International Journal of Network Management* **28**, e1996 (2018).

[7] Wang, X., Chen, M. & Xing, C. Sdsnm: a software-defined security networking mechanism to defend against ddos attacks. In *2015 ninth international conference on frontier of computer science and technology*, 115–121 (IEEE, 2015).

[8] Yuan, B. et al. Defending against flow table overloading attack in software-defined networks. *IEEE Transactions on Serv. Comput.* **12**, 231–246 (2016).

[9] Sahay, R., Blanc, G., Zhang, Z. & Debar, H. Aroma: An sdn based autonomic ddos mitigation framework. *computers & security* **70**, 482–499 (2017).

[10] Hameed, S. & Ahmed Khan, H. Sdn based collaborative scheme for mitigation of ddos attacks. *Futur. Internet* **10**, 23 (2018).

[11] Wang, Y., Hu, T., Tang, G., Xie, J. & Lu, J. Sgs: Safe-guard scheme for protecting control plane against ddos attacks in software-defined networking. *IEEE Access* **7**, 34699–34710 (2019).

[12] Conti, M., Lal, C., Mohammadi, R. & Rawat, U. Lightweight solutions to counter ddos attacks in software defined networking. *Wirel. Networks* **25**, 2751–2768 (2019).

[13] Karmakar, K. K., Varadharajan, V. & Tupakula, U. Mitigating attacks in software defined networks. *Clust. Comput.* **22**, 1143–1157 (2019).

[14] Mishra, A., Gupta, N. & Gupta, B. Defense mechanisms against ddos attack based on entropy in sdn-cloud using pox controller. *Telecommun. systems* **77**, 47–62 (2021).

[15] Shalini, P., Radha, V. & Sanjeevi, S. G. Docus-ddos detection in sdn using modified cusum with flash traffic discrimination and mitigation. *Comput. Networks* **217**, 109361 (2022).

[16] Ahalawat, A., Babu, K. S., Turuk, A. K. & Patel, S. A low-rate ddos detection and mitigation for sdn using renyi entropy with packet drop. *J. Inf. Secur. Appl.* **68**, 103212 (2022).

[17] Wang, J., Wang, L. & Wang, R. A method of ddos attack detection and mitigation for the comprehensive coordinated protection of sdn controllers. *Entropy* **25**, 1210 (2023). 37628240 10.3390/e25081210 PMC10453536

[18] Cui, Y. et al. Sd-anti-ddos: Fast and efficient ddos defense in software-defined networks. *J. Netw. Comput. Appl.* **68**, 65–79 (2016).

[19] Long, Z. & Jinsong, W. A hybrid method of entropy and ssae-svm based ddos detection and mitigation mechanism in sdn. *Comput. & Secur.* **115**, 102604 (2022).

[20] Phan, T. V., Van Toan, T., Van Tuyen, D., Huong, T. T. & Thanh, N. H. Openflowsia: An optimized protection scheme for software-defined networks from flooding attacks. In *2016 IEEE sixth international conference on communications and electronics (ICCE)*, 13–18 (IEEE, 2016).

[21] Sambangi, S., Gondi, L., Aljawarneh, S. & Annaluri, S. R. SDN DDoS Attack Image Dataset, 10.21227/k6q-3t33 (2021).

[22] Ahuja, N., Singal, G. & Mukhopadhyay, D. DDOS attack SDN Dataset, 10.17632/jxpfjc64kr.1 (2020).

[23] Kurniawan, M., Yazid, S. & Sucahyo, Y. G. Comparison of feature selection methods for ddos attacks on software defined networks using filter-based, wrapper-based and embedded-based. *JOIV: Int. J. on Informatics Vis.* **6**, 809–814 (2022).

[24] Liu, Z. et al. A ddos detection method based on feature engineering and machine learning in software-defined networks. *Sensors* **23**, 6176 (2023). 37448025 10.3390/s23136176 PMC10346601

